# Touch and Hearing Mediate Osseoperception

**DOI:** 10.1038/srep45363

**Published:** 2017-03-28

**Authors:** Francesco Clemente, Bo Håkansson, Christian Cipriani, Johan Wessberg, Katarzyna Kulbacka-Ortiz, Rickard Brånemark, Karl-Johan Fredén Jansson, Max Ortiz-Catalan

**Affiliations:** 1 The BioRobotics Institute, Scuola Superiore Sant’Anna, Pisa, Italy; 2 Department of Signals and Systems, Chalmers University of Technology, Gothenburg, Sweden; 3 Institute of Neuroscience and Physiology, University of Gothenburg, Gothenburg, Sweden; 4 Centre for Advanced Reconstruction of Extremities, Sahlgrenska University Hospital, Gothenburg, Sweden; 5 International Center for Osseointegration Research, Education and Surgery (iCORES), Department of Orthopaedics, University of California, San Francisco, USA; 6 Integrum AB, Gothenburg, Sweden

## Abstract

Osseoperception is the sensation arising from the mechanical stimulation of a bone-anchored prosthesis. Here we show that not only touch, but also hearing is involved in this phenomenon. Using mechanical vibrations ranging from 0.1 to 6 kHz, we performed four psychophysical measures (perception threshold, sensation discrimination, frequency discrimination and reaction time) on 12 upper and lower limb amputees and found that subjects: consistently reported perceiving a sound when the stimulus was delivered at frequencies equal to or above 400 Hz; were able to discriminate frequency differences between stimuli delivered at high stimulation frequencies (~1500 Hz); improved their reaction time for bimodal stimuli (i.e. when both vibration and sound were perceived). Our results demonstrate that osseoperception is a multisensory perception, which can explain the improved environment perception of bone-anchored prosthesis users. This phenomenon might be exploited in novel prosthetic devices to enhance their control, thus ultimately improving the amputees’ quality of life.

Sensitivity to vibration has been broadly studied in the past in both health and disease^1^,^2^,^3^,^4^,^5^,^6^. Different hypotheses were proposed, leading to different conclusions about the biological structures and systems responsible for the sensation. In particular, in the late nineteenth century a controversy was raised whether vibration was a discrete sense by its own^7^ or just repeated touch^8^. In line with the first hypothesis, Egger^9^ postulated that the relevant receptors were located in the periosteum and that the bones conveyed information concerning the “shocks of locomotion” and other gross bodily movements.

More recent studies from the 1960 s settled the dispute, identifying the Pacinian corpuscle as the cutaneous receptor which is most sensitive to vibration^1^,^10^,^11^; such specificity was attributed to the mechanical structure of the receptors and how they transmit mechanical stimuli^12^. These biological sensors respond to mechanical vibrations with frequency from 25 to 650 Hz, with a maximum sensitivity in the region 250–350 Hz^1^. However, audiometry studies have shown that bone conduction transducers can evoke a tactile sensation at frequencies up to 1 kHz when used to stimulate the skin over the forehead and mastoid of the temporal bone (albeit substantially larger stimulations are required to elicit such sensations)^13^. In humans, Pacinian corpuscles are particularly dense in the subcutaneous connective tissue, particularly in the fingertips, hand and foot soles^14^. They also spread on several body parts, including fascial planes, joints, pancreas and the periosteum covering the bone^15^, thus supporting the idea of bony sensitivity to vibration as proposed by Egger more than a century ago^9^.

On the other hand, mechanical vibration transmitted through air and through bone in the range of 0.1–10 kHz can also efficiently stimulate the cochlea (maximum sensitivity within the 2–5 kHz range) and thus be clearly perceived as sound^16^.

The two sensory systems involved (i.e. touch and hearing) are intimately interconnected: many sounds can induce sensations that are felt on the body, such as high-pitched screeching noises or low-frequency thumping sounds^17^. Moreover, modifying a stimulus delivered to one of the two sensory systems can greatly affect the perception of the stimulus detected by the other system. This interaction can be specific with respect to the stimulation frequency^18^, but it is also true even during very common tasks, as in the case of the Parchment-skin illusion^19^.

Building on the fact that Pacinian corpuscles cover the bone and that sound can be transmitted through bone as well, in this work we hypothesized that both touch and hearing are involved in the so called phenomenon of osseoperception. This is defined as the sensation arising from the mechanical stimulation of a bone-anchored prosthesis^20^.

This phenomenon has been known for decades, and is believed to play an important role in the rehabilitation of patients receiving dental implants or artificial limbs^21^,^22^. However, only few studies have investigated the phenomenon^23^,^24^,^25^,^26^ and therefore the underlying mechanism(s) responsible for the perception(s) remains a matter of debate^21^. The starting point of past studies was that users of bone-anchored prostheses reported having a subjectively improved ability to “feel” through their prosthesis (i.e. via the anchoring implant in the bone), e.g. improved environment perception, such as type of soil on which they walk^23^ or noticeable improvement in the oral tactile function^21^ with respect to socket-suspended (i.e. supported by the soft tissue of the residual limb through a socket) prosthesis users. For this reason, Jacobs *et al*.^23^ investigated vibration and force perception thresholds in upper and lower limb amputees using both traditional and bone-anchored prostheses. They found that bone-anchored prosthesis users were more sensitive to vibrations than users of socket-suspended ones. However, they delivered stimuli limited in frequency up to 250 Hz, which do not necessarily match the bandwidth of stimuli encountered during activities of daily living. Thus, their study provided a limited view of the phenomenon, arguably restricted to tactile sensations.

For the first time, in the present work we investigated the role of both touch and hearing in osseoperception, specifically when a vibratory stimulus is delivered directly to the bone through an abutment (i.e. the skin-penetrating part of the implant which serves as the anchoring site for the prosthesis; Fig. 1). This study is focused on upper and lower limb amputations as these individuals might benefit from the potential clinical translation of the results. Indeed, this channel could be exploited to convey sensory feedback related to the status of the prosthesis, e.g. hand aperture, grasp force, gait phase.

We examined the osseoperception phenomenon by performing four psychophysical experiments (Fig. 2). In particular, we measured the perception threshold to vibration in the 0.1–6 kHz range (Experiment 1), the bandwidth of the stimulus eliciting different sensations (i.e. tactile, auditory or both, Experiment 2), the subjective frequency discrimination ability for the different elicited sensations (Experiment 3) and the subjective reaction time (Experiment 4).

We expected that, if sound was involved in the phenomenon, individuals would be able to perceive and discriminate among a wider frequency range of stimuli. However, it was unclear which sensation (either tactile or auditory) would lead to better performance during the psychophysical experiments. Indeed, for instance, because of the relatively advantageous position of the mechanoreceptors (spread all over the bone) with respect to the more remote auditory organ (the cochleae are located in the temporal bone of the skull), one would expect that when stimulating the abutment subjects would mainly experience a tactile sensation. Nonetheless, we found that perception thresholds are of the same order of magnitude for both tactile and auditory sensations. In fact, hearing is the predominant sensory system (i.e. auditory perception thresholds are generally lower than tactile thresholds) for a larger portion of the frequency range involved in osseoperception. Our findings provide new insights on the relationships between touch and hearing and open up new possibilities to improve the quality of life of disabled people being rehabilitated with a bone-anchored prosthesis.

## Results

The four psychophysical experiments were performed on twelve subjects rehabilitated with an osseointegrated prosthesis. For data analysis, the subjects were split in two groups depending on the amputation level (Table 1): trans-femoral (lower limb, LL, n = 8) and trans-humeral (upper limb, UL, n = 4). In case that the assumption of normality was verified, a repeated measures analysis of variance (RM-ANOVA) was used. If normality was not verified, the Friedman test was used instead. All subjects participated in all experiments except for subject UL4 who only completed Experiment 1 for reasons unrelated to the study.

### Experiment 1: perception threshold (PT)

The perception threshold at different frequencies (0.1–6 kHz range) was measured using a standard two-interval forced-choice (2IFC) threshold procedure^27^, in which the reference stimulus was the null stimulus and the target stimulus changed in amplitude from trial to trial (Fig. 2) according to a stochastic approximation staircase (SAS^28^).

The upper limit of the osseoperception bandwidth, calculated independently from the perceived modality (tactile or auditory), was found to fall between 750 Hz and 3000 Hz for most of the subjects (Fig. 3, panel B). Only three of them (one LL and two UL amputees) experienced sensations at frequencies above this limit (3000 Hz and 6000 Hz). The lowest perception thresholds were found for the UL group at 100 Hz (Fig. 3, panel B, right). At that frequency they ranged between 0.017N and 0.021N (here and in the following peak to peak values are presented). Higher perception thresholds were found for the LL group at 1500 Hz, with a maximum of 2N. Perception thresholds were found to increase with frequency generally from 750 Hz for both groups.

The RM-ANOVA performed on data from the LL group (Fig. 3, panel B, left) reported a significant effect for the stimulation frequency (F(4,20) = 3.82, p = 0.0182). The post-hoc analysis (Tukey’s honest significant criterion – HSC – correction) revealed a statistical difference between the thresholds at 100, 200 and 400 Hz and the one found at 1500 Hz (p = 0.0354, p = 0.0295 and p = 0.0364 respectively). No statistical difference was found for the UL group (F(4,12) = 2.4418, p = 0.1037).

Subject LL8 (amaranth in Fig. 3, panel B, left) was evidently less sensitive than others. Indeed, he was not able to perceive any stimulus delivered at 100 Hz and above 750 Hz, and perception thresholds at the remaining test frequencies (200 Hz, 400 Hz and 750 Hz) were larger than all other subjects from his group. For this reason, he was removed from the analysis of this particular experiment.

### Experiment 2: sensation discrimination (SD)

In order to know the predominant sensation elicited for each stimulation frequency, subjects were asked to report the type of sensation (tactile, auditory or both) elicited by single stimuli randomly varying in frequency and amplitude (Fig. 2, panel B). Stimuli amplitudes corresponding to a 50% detection level (D_50_) were then compared between sensation types.

D_50_ for tactile sensations (D_50T_) were found to be lower than D_50_ for auditory sensations (D_50A_) in most of the subjects at 100 Hz and 200 Hz (Fig. 4). On the contrary, D_50A_ were lower at 400 Hz and higher stimulation frequencies. Thus, subjects detected tactile stimuli better below 300 Hz and auditory stimuli better above such limit. This was observed in both LL and UL subjects, but reached statistical significance only for the LL group at 100 Hz (binomial probability B(5; 5, 0.5) = 0.0313), 400 Hz (binomial probability B(7; 8, 0.5) = 0.03125), 750 Hz (binomial probability B(8; 8, 0.5) = 0.0039) and 1500 Hz (binomial probability B(5; 5, 0.5) = 0.0313). Interestingly, the participants reported that the stimuli perceived as tactile sensations were localized in the middle of the arm/leg; auditory sensations were instead localized in the skull.

### Experiment 3: frequency discrimination (FD)

As in Experiment 1, the subject’s ability in discriminating stimuli delivered at different frequencies for different elicited sensations (tactile, auditory or both) was measured using a 2IFC threshold procedure where subjects were asked to report which of two stimuli was delivered at higher frequency (Fig. 2, panel C).

For most of the subjects the frequency of the reference stimulus for the tactile (T), tactile and auditory (T+A) and auditory (A) conditions was 100 Hz, 400 Hz and 1500 Hz, respectively, as derived from the outcomes of Experiments 1 and 2. When the stimulus elicited an auditory sensation (T+A and A conditions), the frequency discrimination ability (FDA) of the subjects generally improved (Fig. 5, panel B). In the LL group, the median minimum perceivable frequency difference between the reference and target stimulus reduced from 15.9% (min = 9.2%, max = 554%) in the T condition to 4.15% (min = 0.8%, max = 11.4%) and 7.35% (min = 0.4%, max = 11.6%) in the T+A and A conditions, respectively. In the UL group, it reduced from 104.5% (min = 82.8%, max = 129%) in the T condition to 3.17% (min = 1.4%, max = 3.7%) and 1.41% (min = 0.7%, max = 3.3%) in the T+A and A conditions, respectively.

Statistical difference was found for both the LL group (Friedman test, χ^2^(2) = 8.8571, p = 0.0119) and the UL group (RM-ANOVA, F(2,4) = 63.81, p = 0.0009). In the LL group statistical difference was found between the T and T+A conditions (p = 0.0092); for the UL group the post-hoc analysis revealed a statistical difference between the T and both the T+A and A conditions (p = 0.0014 and p = 0.0013). In both groups, no statistical difference was found between the T+A and A conditions.

### Experiment 4: reaction time (RT)

The last experiment aimed to assess the reaction time to stimuli eliciting different sensations. Results from this experiment followed a similar trend for both groups (Fig. 6): the reaction time for the T+A condition was generally shorter than the reaction time in the other two conditions, followed by the A condition and then by the T condition. In all cases and for all subjects, the median reaction time ranged between 0.3 s and 0.6 s. In particular, results from both groups were largely comparable. The median reaction time for the LL group was 0.46 s, 0.37 s and 0.43 s for T, T+A and A conditions, respectively. The median reaction time for the UL group was 0.57 s, 0.37 s and 0.43 s for T, T+A and A conditions, respectively.

The RM-ANOVA revealed a significant effect of the kind of elicited sensation for both the LL (F(2,14) = 6.6725, p = 0.0092) and UL (F(2,4) = 7.4171, p = 0.0451) groups. In both cases the post-hoc analysis revealed a statistical difference between the T and the TA conditions (p = 0.0078 and p = 0.0404 for the LL and UL group, respectively). No statistical difference was found between the A and the T/T+A conditions (p = 0.46/0.0766 and p = 0.15/0.4 for the LL and UL group, respectively).

## Discussion

In this work, we directly stimulated the implant of 12 amputees fitted with an osseointegrated prosthesis with a mechanical vibration in order to investigate the phenomenon of osseoperception. We hypothesized that the documented improved sensitivity of individuals with osseointegrated prostheses compared to individuals using socket-suspended prostheses could be explained by the combined effect of two sensory systems: touch and hearing. Our results corroborate this hypothesis. In all four experiments we performed, depending on the frequency of stimulation, the participants reported to either feel a vibration or hear a sound when the mechanical transducer was activated, albeit wearing both earplugs and headphones which blocked all air-borne sound from the environment. This was also confirmed by the reported location of the perceived stimulus: at lower frequencies subjects reported to perceive a mechanical vibration in the middle of the arm/leg, while at higher frequencies subjects reported to hear a sound (located in the skull).

Quantitatively, subjects were able to perceive stimuli delivered with frequencies up to 1500 Hz (Fig. 3), which is unlikely if the somatosensory system is taken into account only (Pacinian corpuscles can detect vibrations up to 1 kHz at most^2^,^13^). In addition, all subjects consistently reported hearing for stimuli delivered at 400 Hz or higher frequencies (Fig. 4). The frequency discrimination ability of subjects was also found to be notably good at relatively high frequencies (around 1500 Hz; condition A in Fig. 5). Finally, we observed a reduction in the reaction time when the stimulus was perceived through both sensory modalities at the same time (Fig. 6). All these results provide evidence that not only touch, but also hearing is responsible for the phenomenon of osseoperception.

Our outcomes were based on four independent measures, which we found to converge to results all corroborating our hypothesis. This both represents a methodological strength for this study and underlines the robustness of our results. Another methodological asset of the present study (with respect to previous experiments^23^,^24^) is that established procedures were used, namely the two-interval forced-choice threshold estimation and the stochastic approximation staircase procedure. Such design ensured that subjects compared two stimuli, rather than making a categorical judgment about the perception of the target stimulus. This prevented our results from being *criterion-dependent*, i.e. that different subjects adopt different criteria to assess the strength of a stimulus^27^.

Commonly used statistical methods were used to analyse the results of the study. Albeit useful, in this case the information on statistical significance is limited because of the limited number of patients involved in the study (especially in the UL group). The limited availability of patients having an osseointegrated implant for the attachment of a prosthetic limb limited our possibility to carry out a larger study. For these reasons, we present here a descriptive discussion of the results obtained from the four psychophysical experiments.

Results from Experiment 1 were reported in terms of Newton instead of displacement in contrast to previous studies^1^,^2^. This choice was dictated by the fact that the impedance of the stimulated system (i.e. abutment) is very large when compared to the skin, which was targeted by previous studies. Thus the transducer, vibrating on the rigid abutment and not on the soft skin, imposed to the system a force rather than a displacement.

The subjects were able to perceive stimuli delivered with a wide range of frequencies, i.e. from 100 to 1500 Hz (Fig. 3). The minimum perception thresholds were found to be around 100–200 Hz (Fig. 3), which is in line with the sensitivity of the Pacinian corpuscles in the hairy skin^2^. All subjects reported to perceive a tactile stimulus at those frequencies (Fig. 4). Thresholds for 400 Hz were found to be similar to those at lower frequencies (Fig. 3) but, remarkably, for these frequencies practically all subjects reported perceiving a sound rather than a vibration (Fig. 4). This indicates that the two sensory systems can be accessed by providing similar amount of energy to the abutment. This is unexpected as eliciting an auditory sensation requires the stimulus to travel along several joints and bones in order to stimulate the cochleae in the skull; a path that is assumed to dampen the stimulus amplitude^29^.

Perception thresholds resulted to be lower for the LL group than for the UL group for stimulations at 750 Hz (Fig. 3). Although the larger distance from the cochlea would suggest that a lower limb stimulation should be perceived less, the first transmission path could actually be more efficient. Indeed, even if originated closer, the stimulus from the arm has to travel through several perpendicular turns (shoulder and neck) before reaching the skull. It might thus be that the straighter transmission line (even if longer) through the more robust bones of the spinal cord and the incompressible cerebrospinal fluid entering directly within the skull comes out to be more efficient. Supporting this conclusion is that diagnoses of superior semi-circular canal dehiscence syndrome (SCDS - a pathological “third” window in the bony wall of the superior arc of the balance organ thus communicating directly with the cerebrospinal fluid) are currently performed using a tuning fork applied to the ankle, as this location was found to be fairly good in transmitting the vibration as compared to other locations on the body below the neck^30^.

Experiment 2 proves that the auditory sense is not only involved in the perception of the stimulus, as we hypothesized, but plays a pivotal role. Indeed, from 300–400 Hz up to 1500 Hz the 50% proportion detection levels for auditory sensations were lower than for tactile ones in the majority of the subjects tested.

The frequency discrimination ability of subjects was found to be lower when only a tactile stimulus was perceived: the minimum perceivable frequency difference between the reference and target stimulus was found to be around 16% and 100% the reference frequency for the LL and the UL groups, respectively (condition T in Fig. 5). This is larger compared to previous findings for the UL group, which reported values of ~30% for stimulations delivered at the forearm^31^. A possible explanation for this apparent mismatch could be provided by the fact that there is a longer distance between the source of the stimulus (i.e. the abutment in the middle of the bone) and the biological receptors (on the bony surface) with respect to when the skin is stimulated (the skin of the forearm was stimulated in ref. 31). Performance improved remarkably for higher frequencies. The minimum perceivable frequency difference was found to be lower than 12% the reference stimulus for all subjects when a sound was heard as well (conditions T+A and A in Fig. 5). This is even lower than the frequency discrimination ability at the fingertip for tactile stimulations^31^. This is intuitive as the hearing sensory system of humans is specifically designed to emphasize differences in frequency^16^. On the other hand, such result is unexpected as attenuation of vibration through the bone increases with frequency^29^, while the performance of subjects did not degrade significantly with frequency (i.e. no significant differences were observed between T+A and A conditions). This is important especially from an application side, as frequency modulations over a 1 kHz bandwidth could be used to provide information to amputees through their implants. It has also to be noted that the stimuli were not equated for perceived intensity, and thus subjects could have used intensity cues to discriminate between two stimuli with different frequencies.

Reaction times were generally larger than what was previously reported for vibrotactile and auditory stimulations^32^. This could probably be due to the fact that this experiment was performed at the end of the experimental session (i.e. when the subjects had been already cognitively taxed). However, this was a minor concern as our goal was to compare the different stimulation conditions rather than finding its absolute value. In particular, we observed a shortening in the reaction time when both sensations were elicited by the stimulation (T+A condition), for both the tested groups. This is in line with previous research which showed that bimodal stimuli reduce the reaction time to the stimuli^33^.

Anecdotally, subject LL4 reported referred sensations perceived on her phantom foot when the implant was vibrated. This was the only subject reporting phantom sensations. Subject LL8 was the only subject that underwent an amputation because of infection. Notably, he was also less sensitive to the provided stimuli (amaranth in Fig. 3). This could be due to chemicals routinely used for controlling infections, such as aminoglycosides (which are oto-toxic in higher doses). However, as the amputation was performed in a hospital in another country, it was not possible to access his clinical information.

In this paper, we referred to tactile and auditory sensations generated by the mechanical stimulus applied to the abutment of a bone-anchored prosthesis. However, albeit Pacinian corpuscles covering the bone probably play a major role, the osseopercetion phenomenon is likely to involve the whole somatosensory system, i.e. mechanoreceptors located in the skin and soft tissues, as well as in muscles, joints etc.^20^. In addition, considering our results, the current definition of osseoperception might need to be extended to include the contribution of hearing to the phenomenon. The original definition, which attributed the perception to peripheral somatosensory receptors only^20^, could be modified as follows (added text in italics): “This phenomenon [osseoperception] may be defined as: (i) the sensation arising from mechanical stimulation of a bone-anchored prosthesis, transduced *at lower frequencies* by mechanoreceptors that may include those located in muscle, joint, mucosal, cutaneous and periosteal tissues *and at higher frequencies by the cochlea located in the skull*; together with (ii) a change in central neural processing in maintaining sensorimotor function”.

The pivotal role of hearing in the perception could explain the improved environment perception of amputees treated with osseointegrated prostheses with respect to subjects using traditional socket suspended devices. Additionally, we also found that the interaction of the two sensory channels is additive (Fig. 6), leading to improved performance of subjects when the stimulus is perceived through both sensory channels. This could be exploited in novel prosthetic devices able to deliver sensory feedback, enhancing the sense of ownership of the prosthesis and improving the quality of life of the amputees. This being said, it must be noted that the notion of osseoperception as a multisensory perception still requires a great deal of elaboration and we expect that future empirical and modelling studies will investigate the contributions of touch and hearing to the phenomenon in more detail. For instance, it is an open question to what extent such multi-modal perception influences the amputees’ way of using and interacting with their own prosthesis during activities of daily living. Additionally, it would be interesting to investigate the interactions between touch and hearing, and how each sensation affects the perception of the other.

## Methods

Twelve (upper and lower) limb amputees (all naïve to psychophysical procedures) with different amputation levels were enrolled in the study (Table 1). All amputees had an osseointegrated implant and underwent a two-stage surgical procedure in which the fixture is first implanted in the bone, and three to six months later a percutaneous component (the abutment) is coupled to the fixture once this has been osseointegrated^34^,^35^. Our experimental session took place at least 6 months after the second surgery was performed in order to ensure sufficient mechanical stability between the implant (fixture) and the bone. Informed consent in accordance with the Declaration of Helsinki was obtained before conducting the experiments from each subject. The study was approved by the Regional Ethical Review Board in Gothenburg (#802–15). The methods were carried out in accordance with the approved guidelines.

**Figure 1:**
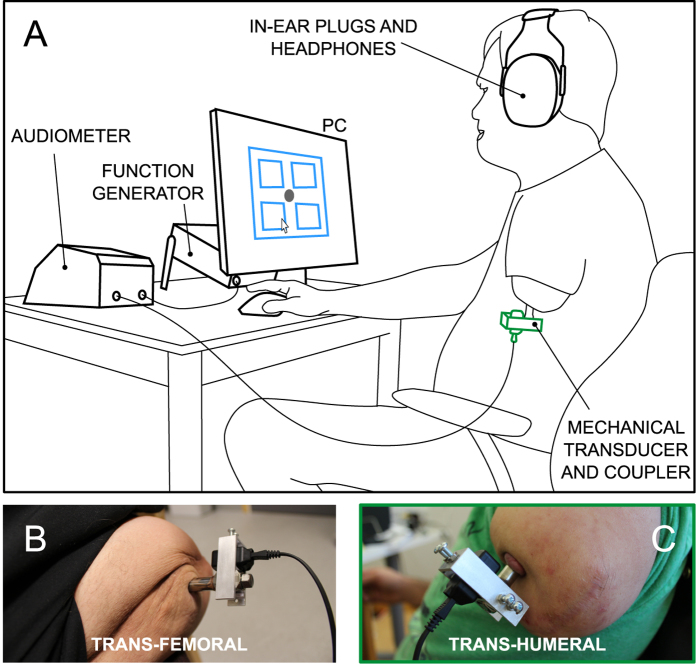
Experimental setup. (**A**) an upper limb amputee performing the sensation discrimination experiment (Experiment 2). The function generator generates a sinusoidal excitation signal that, after being amplified by the audiometer, is converted to a mechanical vibration by the transducer and used to stimulate the user through the titanium implant fixed into the bone. The user interacts with a PC through a user interface which depends on the experiment being performed. Headphones and deep inserted earplugs are used to block any auditory noise eventually generated by the transducer during operation. (**B–C**) the mechanical transducer and coupler are attached transversally to the abutment of the subjects with the two amputation levels tested.

The experimental setup consisted of a function generator (AFG-2112, GW Instek, New Taipei, Taiwan), an audiometer (AC40, Interacoustics, Eden Prairie, MN), a mechanical bone conduction transducer (Radioear B81, New Eagle, PA), a PC, earplugs and headphones (Fig. 1, panel A). The B81 transducer is an improved version of the Radioear B71, and is characterized by having a considerably lower harmonic distortion than previous models (for more details see Fredén Jansson *et al*.^36^).

The transducer was attached to the abutment of the subjects through a custom mechanical coupler, specifically designed to provide a stiff and stable mechanical connection with abutments of different geometry (Fig. 1, panels B and C). The frequency response of the system (vibrator and coupler) was measured using a skull simulator^37^, both before and after the end of the study in order to ensure stability over time. The frequency response was also used to calibrate the bone vibrator in order to calculate the resulting stimulating force.

The function generator was used to generate a sinusoidal excitation signal that, after being amplified by the audiometer, was converted into a mechanical vibration by the transducer and used to stimulate the user. By running a custom software written in C (Labwindows/CVI, National Instruments, Austin, TX, USA), the PC was used to set the frequency and amplitude of each stimulation, run the experimental protocol and provide the subjects with a user interface to interact with during the experimental session. The delay introduced by this setup (i.e. from the moment when the activation command was sent to the audiometer by the PC to the moment when the transducer was activated) was 100 ms.

The subjects were seated in front of a PC mouse and screen, wearing both earplugs and headphones in order to block any auditory noise eventually generated by the transducer during operation (Fig. 1) that could have affected the results at frequencies around 4 kHz^38^. Previous studies showed that this setup provides a signal attenuation level of 40–55 dB in the 0.1–10 kHz frequency range^39^. As an additional safety margin available in the current setup, the transducer was directly attached to a stiff abutment with respect to the soft skin. The stiffer mechanical connection implies that, as the load is larger, the amplitude of the generated vibration is smaller, leading to lower sound emission levels. Care was also taken to ensure that earplugs were inserted deeply in both ear canals in order to avoid the occlusion effect^40^. Finally, pilot studies ensured that this setup effectively masked all auditory sound generated by the transducer. In these tests the experimental setup was reproduced by attaching the B81 transducer to an abutment similar to the ones implanted in the patients participating in the study. Within this setup, when the transducer was driven at signal levels similar to the ones used during the study, no signal was heard.

**Figure 2:**
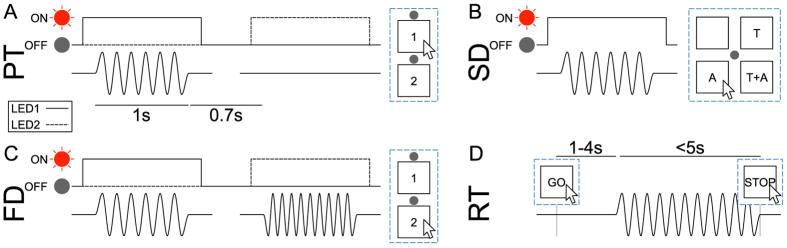
Timing of stimulation and user interface (blue dashed line) for the four psychophysical experiments performed by the subjects. (**A**) (Experiment 1): *Perception Threshold (PT)*. Subjects were asked to detect when a stimulus was delivered, discriminating against the null stimulus. (**B**) (Experiment 2): *Sensation Discrimination (SD)*. Subjects were asked to report which sensation (not perceived, tactile – T –, auditory – A –, or both – T+A) was elicited by each stimulus received. (**C**) (Experiment 3): *Frequency Discrimination (FD)*. Subjects were asked to report which one of two stimuli was delivered with higher frequency. (**D**) (Experiment 4): *Reaction Time (RT)*. Subjects were asked to respond to a stimulus as quickly as possible. In RT, the retention interval was set to randomly vary between 1 s and 4 s. In PT, SD and FD the stimuli were 1 s long. In PT and FD the inter-stimulus interval was set to 0.7 s.

**Figure 3:**
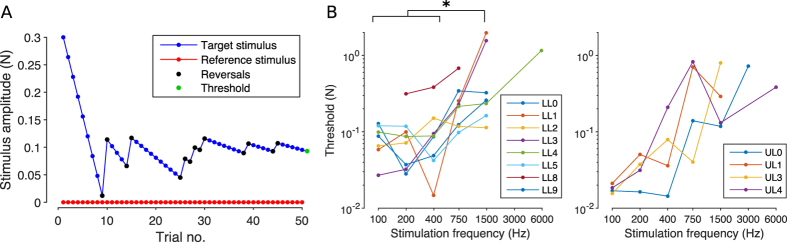
Perception threshold for the two tested groups. (**A**) For each test frequency, the subject performed 50 trials changing the amplitude of the target stimulus according to a stochastic approximation staircase (SAS, see text) targeting 85% detection threshold. The perception threshold was chosen as the amplitude of the 51^th^ stimulus as calculated from the SAS. (**B**) Generally, the osseoperception bandwidth is 100–1500 Hz, with 3 out of 12 subjects perceiving the stimuli at 3 and 6 kHz as well. The lowest perception thresholds (i.e. the maximum sensitivity) were found for the upper limb amputees (UL, right) at 100 Hz, being around 20 mN peak to peak. LL8 was excluded from the statistical analysis as identified as an outlier. *indicates p < 0.05.

**Figure 4:**
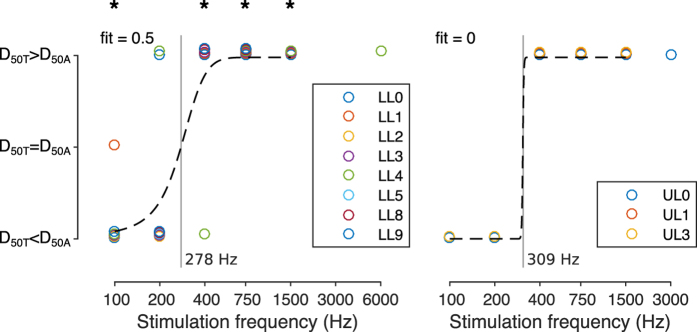
Comparison of 50% detection level for tactile (D_50T_) and auditory (D_50A_) sensations. Tactile sensations were elicited by lower amplitude stimuli at 100 Hz and 200 Hz (D_50T_ < D_50A_) in both groups. On the contrary, lower amplitude stimuli elicited auditory sensations at 400 Hz and higher frequencies (D_50T_ > D_50A_). *indicates p < 0.05 for a specific stimulation frequency.

**Figure 5:**
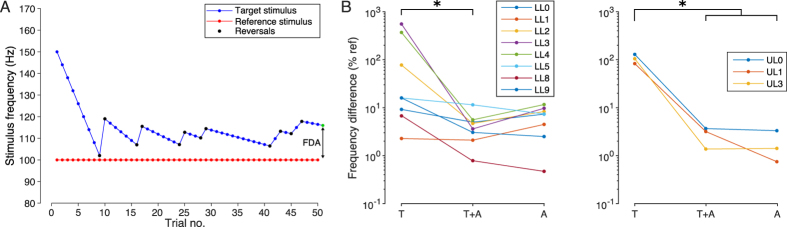
Frequency discrimination ability as a function of the sensation elicited by the stimulus. (**A**) As in Experiment 1, a SAS was used to calculate at each trial the frequency of the target stimulus, and the frequency discrimination ability (FDA) of subjects was calculated as the difference in frequency between the reference stimulus and the target stimulus that would have been presented on the 51^th^ stimulation. (**B**) The median frequency difference between the reference and target stimulus that subjects were able to discriminate (i.e. the just noticeable frequency difference) ranged between 1.41% for the upper limb (trans-humeral, UL, right) group in the A (auditory) condition and 104.5% for the lower limb (trans-femoral, LL, left) group in the T (tactile) condition. Generally, the ability of subjects in discriminating among stimuli with different frequency improved when sound was involved in the perceived sensation (tactile and auditory – T+A – and A conditions). In these cases, for the LL group, the perceivable frequency difference between the reference and target stimulus was 4.15% (min = 0.8%, max = 11.4%) and 7.35% (min = 0.4%, max = 11.6%) in the T+A and A conditions, respectively, and 3.17% (min = 1.4%, max = 3.7%) and 1.41% (min = 0.7%, max = 3.3%) for the UL group. *Indicates p < 0.05. LL and UL indicate the level of amputation, respectively lower limb (trans-femoral) and upper limb (trans-humeral). For most of the subjects the frequency of the reference stimulus for the T, T+A and A conditions were 100 Hz, 400 Hz and 1500 Hz, respectively.

**Figure 6:**
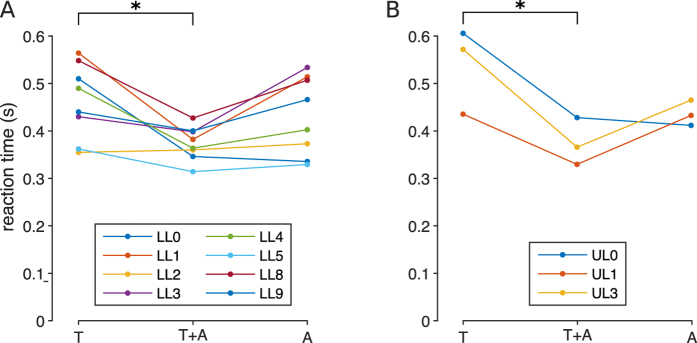
Reaction time as a function of the sensation elicited by the stimulus. Subjects reacted faster to the provided stimulus when both tactile and auditory sensations were perceived (T+A condition), followed by the case in which only auditory sensation was perceived (A condition). Subjects were slowest when a tactile sensation was perceived only (T condition). *indicates p < 0.05. LL and UL indicate the level of amputation, respectively lower limb (trans-femoral, panel A) and upper limb (trans-humeral, panel B). For most of the subjects the frequency of the reference stimulus for the T, T+A and A conditions were 100 Hz, 400 Hz and 1500 Hz, respectively.

Four psychophysical experimental procedures, described below, were implemented: Experiment 1, perception threshold (PT), Experiment 2, sensation discrimination (SD), Experiment 3, frequency discrimination (FD), and Experiment 4, reaction time (RT). At the beginning of each experiment (performed in the same order as presented above), subjects received written instructions from the experimenter on how to perform the upcoming experiment and how to interact with the user interface. Oral instructions were only given if the patient specifically asked for clarifications. In Experiments 1, 2 and 3 the mechanical stimuli delivered were sinusoidal vibrations with 1.0 s duration and varying frequency and amplitude (depending on the experiment; Fig. 2). In Experiment 4, the stimulus duration was dependent on the user (see description below). The rising and falling time of all stimuli was 100 ms. The transients were automatically generated by the audiometer, which is specifically designed in order to minimize signal bounces.

### Experiment 1: perception threshold (PT)

This experiment aimed at measuring, for different frequencies, the amplitude perception threshold (i.e. the minimum perceivable stimulus) of subjects when the implant is vibrated.

A standard two-interval forced-choice (2IFC) threshold procedure was used^27^: on each trial the subjects are asked to compare two consecutive stimuli delivered to their abutment, separated by a given retention interval. For the PT experiment (Fig. 2, panel A), one of the stimuli was the null stimulus and the other, i.e. the *target stimulus*, changed in amplitude from trial to trial according, in our case, to a *stochastic approximation staircase* (SAS, described below).

The SAS was used to calculate the amplitude of the target stimulus, according to the following equation:


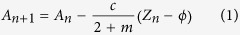


where:

- A_n_ is the amplitude of the target stimulus during the previous trial;
- c is a constant (depending on the subject, see below);
- m is the number of reversals (how many times the answer changes from wrong to right or vice-versa);
- *ϕ* is the stimulus detection probability threshold to which the staircase should converge (set to 0.85);
- Z_n_ is set to 1 if the answer is right, and to 0 if wrong.

With respect to other methods, the SAS has the advantage of automatically adjusting the step size 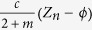 depending on the user answer *Z*_*n*_ and the number of reversals *m*. This tailors the task difficulty to individual performance, making the test less vulnerable to ceiling or flooring effects, and keeping its difficulty consistent across all experimental conditions^41^.

In order to obtain a rough estimate of the threshold to be used as a handle on the subsequent SAS, the method of limits was used before the test^27^: two ascending and two descending series were performed, and the resulting threshold (averaged among the four series) was multiplied by 3 and used as the *c* parameter. According to equation (1), the amplitude of the next stimulus was thus increased if the answer was incorrect (i.e. if the subject selected the null stimulus), while it was decreased if correct. The subjects were never given feedback on their response.

During the whole experiment, the subjects interacted with a graphical user interface on the PC screen comprised of two LEDs and two numbered buttons (each of which associated spatially with one LED; upper left inset in Fig. 2). While one of the two stimuli was delivered, one of the two LEDs turned on. At the end of the second stimulus, the subject was instructed to click on the button corresponding to the LED associated with the target stimulus (the association between LEDs and target/null stimulus was randomized). In other words, as one of the two stimuli was actually the null stimulus, subjects were basically asked to detect *when* they perceived the (target) stimulus.

A single trial consisted of delivering both the null and target stimuli (in random order), and allowing the user to consequently select one of the two buttons through the user interface, triggering the beginning of the next trial (Fig. 2, panel A). In this experiment, no indication on the kind of elicited sensation was provided: subjects had to detect the reference stimulus, regardless of the sensation they felt.

The test was repeated once for each test frequency, randomly selected by the software within the following set: 100, 200, 400, 750, 1500, 3000, 6000 Hz. A priori, this set of frequencies was considered to be balanced with respect to the elicited sensation, 100–400 Hz being in the tactile domain (i.e. perceived mainly as mechanical vibration), 1500–6000 Hz being in the auditory domain (i.e. perceived mainly as sound) and 750 Hz being in between^2^,^13^,^16^.

Each test (i.e. each SAS) was stopped after 50 trials, and the value of the stimulus that would have been presented as the 51^st^ trial was chosen as the perception threshold. This method was demonstrated to be more accurate than averaging among a certain number of staircase reversals^28^.

### Experiment 2: sensation discrimination (SD)

In order to understand what kind of sensation was elicited by the stimuli at different frequencies, subjects were presented with a single stimulation and asked to report whether it elicited a tactile sensation (T), an auditory sensation (i.e. if they heard a sound; A) or both at the same time (T+A) by clicking on the corresponding button on the user interface (Fig. 2, panel B). The stimulation frequency was randomly chosen for each stimulus from the same set used in Experiment 1. The stimulation amplitude was randomly chosen among 9 levels spanning across the whole output range of the transducer. Each pair of parameters was presented to the subjects twice, for a total of 126 stimulations.

For each subject, the resulting data was grouped by reference frequency, and a logistic psychometric function (guessing rate γ and lapse rate λ being fixed at 0 and 0.02 respectively) was fitted to the data separately for each sensation type. The stimulus amplitude value corresponding to a 50% proportion detected stimuli (D_50_) was compared between sensation types^27^. The sensation (tactile or auditory) having a lower D_50_ was determined to be the predominant sensation at that frequency.

### Experiment 3: frequency discrimination (FD)

This experiment aimed at measuring the subject’s ability to discriminate between different frequencies based on the elicited sensation. In other words, it measures the just noticeable frequency difference. Three frequencies were selected depending on the output of Experiment 2: one frequency at which only a tactile vibration was felt (condition T), one at which both sensations were reported (condition T+A) and one at which sound was heard (condition A). The amplitude of the stimulus was set to be equal to three times the threshold calculated during Experiment 1 in order to ensure clear stimulus perception. This procedure was already used in past studies, and quickly ensured that the perceived amplitudes were similar across the tested frequencies^31^.

The frequency sensitivity was then calculated using a protocol similar to Experiment 1. In this case the null stimulus was replaced by a *reference stimulus*. In particular, the amplitude of both the reference and target stimuli was the same, while the frequency of the reference stimulus was fixed to the reference frequency and the frequency of the target stimulus was calculated for each trial using equation (1) (Fig. 2, panel C). The frequency of the target stimulus was always larger than the one of the reference stimulus. It should be noted that when the frequency of a vibrotactile stimulus is changed, there can be, and often is, a change in perceived intensity. For this reason, investigators typically equate stimuli in perceived intensity and may present several different levels of intensity in order to render that cue ambiguous^31^,^42^. However, as we were not interested in how the subjects discriminated between two different frequencies (whether on the basis of perceived “pitch” differences or intensity differences), we did not equate the stimuli for perceived intensity as this would have increased the duration of the study and affected the performance of the subjects.

### Experiment 4: reaction time (RT)

The last experiment performed aimed at measuring the reaction time to stimuli eliciting different sensations. The same three reference frequencies from Experiment 3 were selected, while the amplitude of the stimulus was set to be three times the threshold calculated during Experiment 1. The subjects were presented with a stimulus at which they had to respond as quickly as possible by clicking on a button on the user interface (Fig. 2, panel D). The next stimulus was triggered by a second click of the subject, and was delivered after a retention interval randomly varying between 1 s and 4 s. This allowed experimenters to avoid receiving answers from participants based on temporal prediction without affecting the measured reaction time^32^,^43^. If the patient clicked the button before the stimulus was delivered, this was signalled to the subject through the user interface.

The test was repeated 30 times for each reference frequency and the median reaction time was taken as the subject’s reaction time at that specific stimulation frequency. Before conducting the analysis, the 100 ms delay introduced by the setup was subtracted from the data.

### Data analysis

The subjects were split up in two groups, depending on the amputation level: LL (lower limb, trans-femoral amputation – 8 subjects) and UL (upper limb, trans-humeral amputation – 4 subjects). The data was thus analysed separately for each group.

Perception thresholds measured during Experiment 1 are reported in Newton peak to peak. For Experiments 1, 3 and 4, in case that the assumption of normality was verified through the Shapiro-Wilk test, a repeated measures analysis of variance (RM-ANOVA; factor: reference frequency for Experiment 1 or elicited sensation for Experiments 3 and 4) was used. If normality was not verified, the Friedman test was used instead. Hence, if the analysis resulted to be significant, a post-hoc analysis with Tukey’s honest significant criterion was performed.

For Experiment 2, we computed the probability of the observed occasions in which the stimulus was perceived as a vibration at lower amplitudes than as a sound, in all subjects assuming a binominal distribution, i.e. B(x; n,p) with x equal to the number of successes, n equal to the number of subjects in the group and p = 0.5 (i.e. same probability of the two events).

In all cases a p-value of 0.05 was considered statistically significant.

## Additional Information

**How to cite this article:** Clemente, F. *et al*. Touch and Hearing Mediate Osseoperception. *Sci. Rep.* **7**, 45363; doi: 10.1038/srep45363 (2017).

**Publisher's note:** Springer Nature remains neutral with regard to jurisdictional claims in published maps and institutional affiliations.

## Figures and Tables

**Table 1:** Details of participants.

Subject	Gender, Age	Amputation level (Laterality)	Cause of amputation	Years since amputation/Years since second surgery*
LL0	M, 75	Trans-femoral (L)	Trauma	29/16
LL1	M, 49	Trans-femoral (L)	Trauma	2/0.6
LL2	F, 42	Trans-femoral (R)	Trauma	6/1
LL3	M, 66	Trans-femoral (R)	Trauma	8/6
LL4	F, 31	Trans-femoral (L)	Trauma	2/2
LL5	M, 61	Trans-femoral (R)	Tumour	19/16
LL8	M, 73	Trans-femoral (L)	Infection	10/1
LL9	M, 33	Trans-femoral (R)	Trauma	6/3
UL0	M, 43	Trans-humeral (R)	Trauma	12/6
UL1	M, 73	Trans-humeral (L)	Trauma	17/10
UL3	M, 48	Trans-humeral (R)	Tumour	31/19
UL4	M, 43	Trans-humeral (R)	Trauma	6/4

*The osseointegration surgical procedure is usually performed in two steps^35^.
